# Draft Genome Sequence of *Verrucosispora* sp. Strain CWR15, Isolated from a Gulf of Mexico Sponge

**DOI:** 10.1128/MRA.00176-20

**Published:** 2020-03-26

**Authors:** Sarah J. Kennedy, Eleonora Cella, Mohammad Jubair, Taj Azarian, Bill J. Baker, Lindsey N. Shaw

**Affiliations:** a Department of Cell Biology, Microbiology and Molecular Biology, University of South Florida, Tampa, Florida, USA; b Burnett School of Biomedical Sciences, University of Central Florida, Orlando, Florida, USA; c Department of Chemistry, University of South Florida, Tampa, Florida, USA; Georgia Institute of Technology

## Abstract

Here, we present the draft genome sequence of Verrucosispora sp. strain CWR15, a bacterial symbiont of a Gulf of Mexico sponge. The genome consists of 35 contigs encoding 5,840 genes. The genome is the basis for future study and presents an underexplored taxonomy and biosynthetic potential.

## ANNOUNCEMENT

Rheims et al. (1) established the genus Verrucosispora in 1998, and 10 distinct species have been identified to date. Successful culturing of these environmentally and taxonomically rare (2) organisms has resulted in important natural product discoveries, with >18 novel compounds thus far documented, all with various bioactivities (3); these include thiocoraline A (4), androgen agonistic gifhornenolone A (5), and a newly described anti-influenza A compound, abyssomicin Y (6).

*Verrucosispora* CWR15 was isolated from a Gulf of Mexico sponge collected from Clearwater Reef, Florida, in 2015 and cryopreserved in glycerol prior to processing. Sponge tissue was initially macerated in an equal sample mass/volume ratio of filter-sterilized (0.22 μm; MilliporeSigma) Instant Ocean sea salt solution (IOSSS; Aquarium Systems, Inc.). This was then macerated again in 5 volumes of sterile IOSSS and used as a supplementation to M1_low_ agar (2 g starch, 0.8 g yeast extract, 0.4 g peptone, 18 g agar, and 36 g IOSSS per liter deionized water) (7) at a final concentration of 10% prior to being poured as plates and incubated for 4 months at room temperature.

A highly pigmented colony was recovered and then cryopreserved and/or subpassaged multiple times to yield domesticated *Verrucosispora* sp. strain CWR15 (preliminary 16S rRNA gene sequencing). This strain was grown for 1 week (30°C, 210 rpm) in tryptic soy broth (Teknova) supplemented with sterile glass beads and 50% sucrose (wt/vol) solution to a final concentration of 20% (vol/vol) prior to DNA extraction and purification using a DNeasy blood and tissue kit following the manufacturer’s instructions (Qiagen).

Using an Oxford Nanopore MinION instrument, ligation sequencing kit, and R9.4.1 flow cell, we obtained 105.6 Mbp of sequence with a mean read length of 4,488.8 bp (longest read, 54.9 kbp) and a mean read quality of 11.6. Reads were filtered using Filtlong v0.2.0 with the settings min_length 1000 and keep_percent 90. Quality was assessed using NanoPlot v1.0.0. An Illumina MiSeq instrument with a Nextera Flex library preparation kit and V3 flow cell chemistry produced 369.8 Mbp of paired-end 2 × 250-bp reads (>50× idealized coverage). Illumina data were filtered using Trimmomatic v0.39 using the settings SLIDINGWINDOW:10:20 MINLEN:31 TRAILING:20. Unicycler v0.4.8 with default settings (8) was used to resolve a final genome of 35 contigs with a total length of 6,367,494 bp (*N*_50_, 666,828 bp; largest contig, 1.09 Mbp). The SpeciesFinder k-mer tool from the Center for Genomic Epidemiology (https://cge.cbs.dtu.dk/services/KmerFinder/) was used to identify the closest matching reference genome.

Contigs were annotated using the NCBI Prokaryotic Genomes Annotation Pipeline and deposited in the GenBank database. The genome consists of 5,231 protein-coding sequences, 49 tRNAs, and 2 each of the 5S, 16S, and 23S rRNAs. The G+C content is 71%, and the average nucleotide identity to all NCBI-available *Verrucosispora* sp. reference genomes is <92% (Verrucosispora sediminis [GenBank accession number FOLJ00000000], calculated according to Yoon et al. [9]), indicating species demarcation (10). Further demarcation is evident by the average amino acid identity of *Verrucosispora* sp. strain CWR15, which is <93.9% similar to NCBI-available genomes/proteomes (Verrucosispora sediminis [accession number FOLJ00000000], calculated according to Medlar et al. [11]) (12).

### Data availability.

This whole-genome shotgun project has been deposited at DDBJ/ENA/GenBank under the accession number SAIY00000000. The version described in this paper is version SAIY01000000. The SRA accession number is PRJNA512368.
